# Supplementary figures and images for: The Development of 3D Bovine Intestinal Organoid Derived Models to Investigate Mycobacterium Avium ssp Paratuberculosis Pathogenesis
Source: Front Vet Sci. 2022 Jul 4;9:921160. doi: 10.3389/fvets.2022.921160 (PMC9290757; doi:10.3389/fvets.2022.921160)

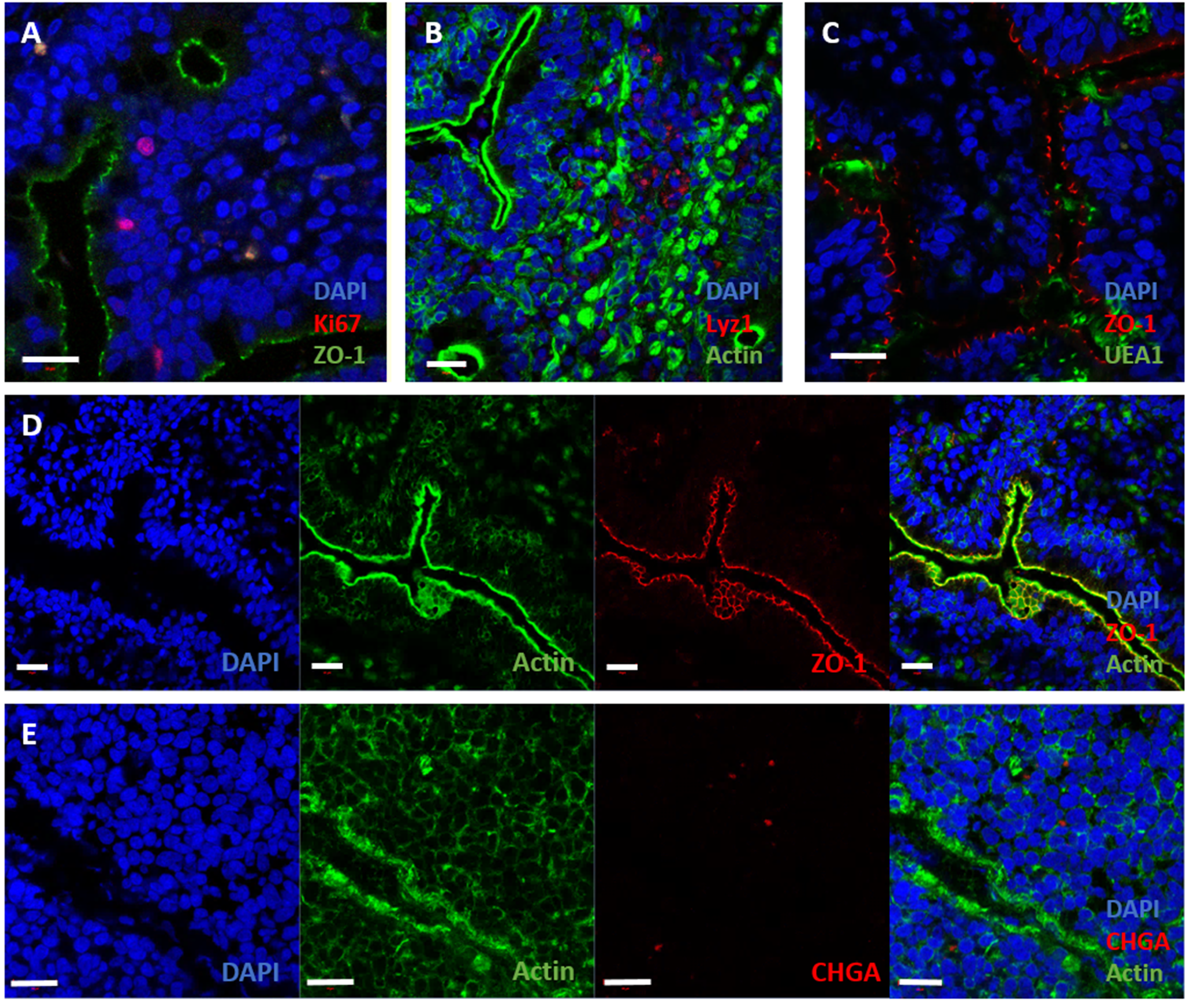

Supplement: Supplementary Figure 1 — Immunofluorescence staining of bovine intestinal tissue slices. Representative confocal images from 2 calves showing staining for epithelial cell fate markers. (A) Tissue slices stained for nuclei (DAPI, blue), tight junctions (ZO-1, green) and proliferative cells (Ki-67, red). (B) Tissue stained for nuclei (DAPI, blue), actin (Phalloidin, green) and Paneth cells (lysozyme, red). (C) Tissue stained for nuclei (DAPI, blue), tight junctions (ZO-1, red) and glycolipids (UEA-1, green). (D) Split panel of tissue stained for nuclei (DAPI, blue), actin (Phalloidin, green), and tight junctions between cells (ZO-1, red). (E) Split panel of tissue stained for nuclei (DAPI, blue), acting (phalloidin, green) and enteroendocrine cells (chromogranin A, red). Scale bar = 20 μm. [file Image_1.TIF]

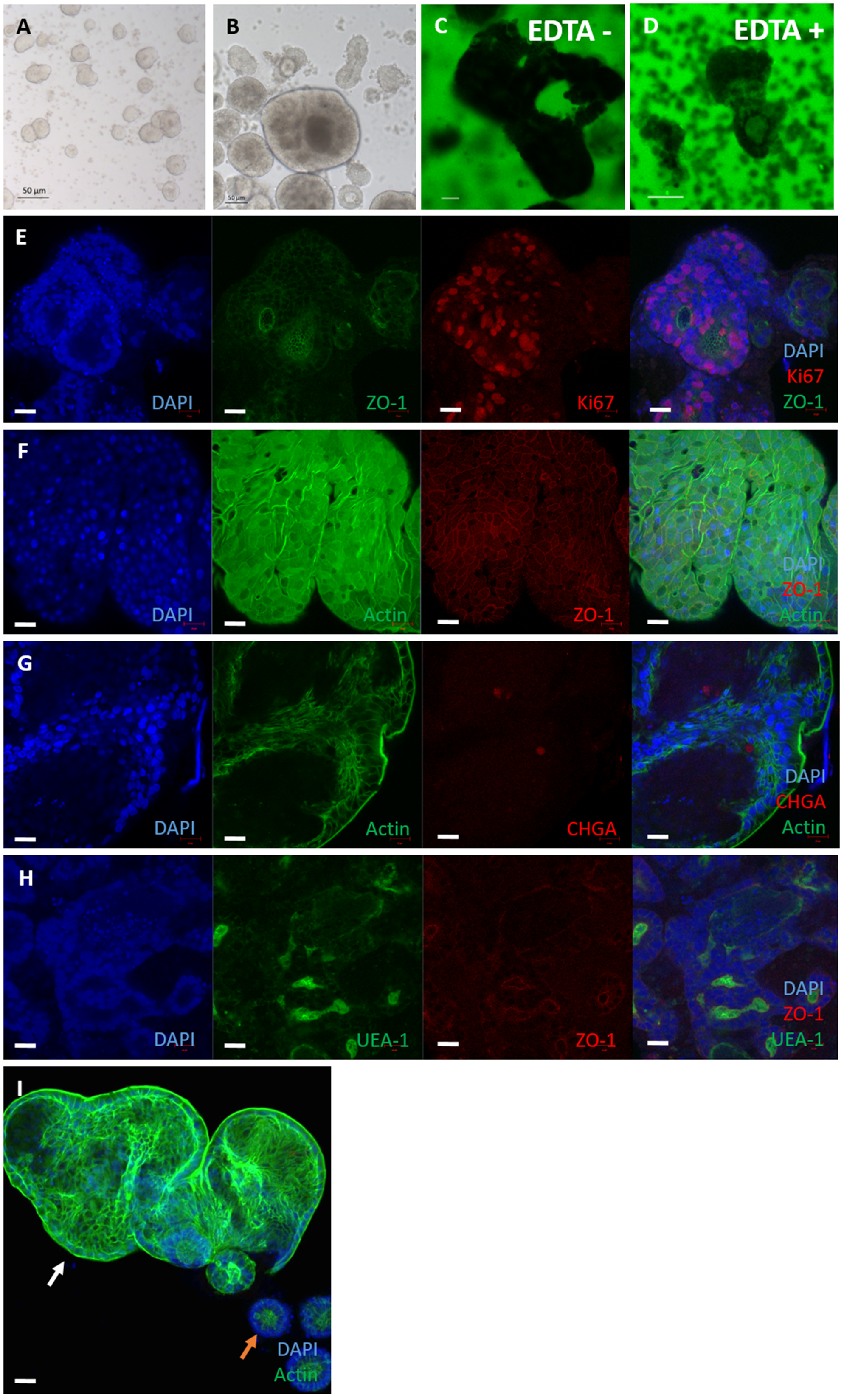

Supplement: Supplementary Figure 2 — Apical-out bovine enteroids show epithelial barrier integrity when established from previously passaged 3D enteroids. Apical-out enteroids established from previously passaged basal-out 3D enteroids at 1 day (A) and 7 days (B). Images are representative of enteroids generated from 1 calf at passage number 5, 11 and 13. (C,D) Confocal images of bovine apical-out enteroids (7 days of culture) immersed in FITC-dextran 4kDa showing epithelial barrier integrity in untreated (C) and EDTA-treatment (D). Scale bar = 50 μm. Immunofluorescence staining of bovine apical-out enteroids shown in split panel demonstrates epithelial differentiation (E–H). Apical-out enteroids were stained for nuclei (DAPI, blue), proliferative cells (Ki67, red), and tight junctions between cells (ZO-1, green) (E). Apical-out enteroids were stained for nuclei (DAPI, blue), tight junctions (ZO-1, red), and actin (Phalloidin, green) (F). Apical-out enteroids stained for nuclei (DAPI, blue), actin (Phalloidin, green) and enteroendocrine cells (Chromogranin A, red) (G). Apical-out enteroids stained for nuclei (DAPI, blue), glycolipids (UEA-1, green) and tight junctions (ZO-1, red) (H). Apical-out enteroids and basal-out 3D enteroids shown to be present in the same culture when generated from previously passaged enteroids (Phalloidin, green and DAPI, blue) (I). White arrow denotes apical-out enteroid, orange arrow denotes basal-out enteroid. Scale bar = 20 μm. [file Image_2.TIF]
