# Supplementary material for: The Development of 3D Bovine Intestinal Organoid Derived Models to Investigate Mycobacterium Avium ssp Paratuberculosis Pathogenesis
Source: Front Vet Sci. 2022 Jul 4;9:921160. doi: 10.3389/fvets.2022.921160 (PMC9290757; doi:10.3389/fvets.2022.921160)
Supplement: Supplementary file 3 [file Table_1.docx]

| **Antibody/ cell stain** | **Concentration for use in confocal microscopy** | **Supplier name and product code** |
| --- | --- | --- |
| Anti-Ki-67 | 10 µg/mL | Abcam, ab15580 |
| Anti-ZO-1 | 5 µg/mL | Thermo Fisher, 1A12 |
| Anti-Lysozyme 1 | 10 µg/mL | Dako, A0099 |
| Anti-Chromogranin A | 20 µg/mL | Santa Cruz biologicals, SC-271738 |
| *Ulex Europaeus* Agglutinin 1 | 10 µg/mL | Vector Laboratories, FL-1061 |
| Anti-MAP | 3 µg/mL | Gene Tex, GTX82978 |
| Phalloidin 488 | 66 µM | Thermo Fisher, A12379 |
| Anti-Rabbit Ig 488 | 10 µg/mL | Thermo Fisher, A11034 |
| Anti-Rabbit Ig 594 | 10 µg/mL | Thermo Fisher, Z25307 |
| Anti-Mouse Ig 488 | 10 µg/mL | Thermo Fisher, A21200 |
| Anti-Mouse Ig 568 | 10 µg/mL | Thermo Fisher, A11004 |
| Anti-Guinea Pig Ig 594 | 10 µg/mL | Sigma Aldrich, SAB4600080 |

**Supplementary Table S1| Table of cell staining reagents for confocal microscopy.**
